# Mentalizing Emotions and Social Cognition in Bullies and Victims

**DOI:** 10.3390/ijerph19042410

**Published:** 2022-02-19

**Authors:** Maria Luisa Pedditzi, Roberta Fadda, Tricia Striano Skoler, Loredana Lucarelli

**Affiliations:** 1 Department of Pedagogy, Psychology, Philosophy, University of Cagliari, 09124 Cagliari, Italy; robfadda@unica.it (R.F.); llucarelli@unica.it (L.L.); 2 Department of Psychology, Hunter College of the City University of New York, New York, NY 10065, USA; tstriano@hunter.cuny.edu

**Keywords:** theory of mind, mentalization, bullying, emotional competence, social cognition

## Abstract

Mentalizing is the ability to represent mental states to navigate the social world. A reduced mentalizing ability is a risk factor for a variety of psychological issues. Several studies indicated deficits in social cognition in bullies and victims, specifically in mentalizing anger. However, only a few studies investigated mentalizing abilities related to both anger and happiness in pre-adolescence. Our study investigated possible differences in the ability to mentalize anger and happiness in preadolescent bullies and victims, compared to a control group. We interviewed 104 preadolescents (44% males; 56% females; M = 13.2 years; SD = 0.82) and administered the Olweus Questionnaire to identify bullies and victims. We applied a narrative approach to investigate the mental state language referred to anger and happiness. The results indicated a reduced ability to mentalize anger in bullies and victims compared to controls. Both bullies and victims tended to consider anger and happiness predominantly as behavioral conditions rather than a state of mind. These results highlight the need to promote effective intervention programs to prevent bullying by enhancing appropriate mentalization of emotions in pre-adolescents.

## 1. Introduction

Mentalizing is the ability to think about one’s own and others’ mental states [1]. It is a mental activity that allows the perceiving of human behavior in terms of mental states like needs, desires, feelings, beliefs, and goals [2,3]. Mentalizing is the reflective function of Theory of Mind, which might be conceived in terms of “representation of subjectivity” [4]. Originally, the term Theory of Mind referred only to epistemic mental states, especially to false beliefs [5,6]. Later, researchers referred to a broader definition of the Theory of Mind, which includes different mental states like emotions and the role of emotions in self-regulation [7].

The ability to mentalize emotions is a component of a broader emotional competence. It allows obtaining access to emotions as affective mental states. It promotes an understanding of interpersonal behavior and self-regulation [3].

According to Saarni [8], emotional competence includes both either the “emotional awareness” and the “ability to use the emotional vocabulary”, which consist in the ability to use mental state terms typical of each culture. In these terms, the investigation of the emotional competence might be extended beyond infancy.

Some studies investigated the ability to mentalize emotions at the beginning of adolescence.

These studies confirm the framework of the hierarchical model of Pons, Harris e de Rosnay [9], which highlights the developmental transition occurring between 3 years old and 11 years old, from a behavioral understanding of emotions to a more advanced level of mentalization, namely the reflective understanding of emotions.

Other studies considered the role of education and individual differences in the development of emotional competence from a developmental perspective [10,11] and a clinical perspective [2]. These studies highlighted the key role of the parents’ mentalization to promote children’s social and emotional development. However, the relationship between the parental ability to mentalize and the development of children’s emotional competence is not so linear. Parental mentalization might promote the understanding of some emotions. At the same time, it might limit the understanding of others, due to possible differences in the familiar and in the cultural framework. Thus, it is important to consider the narration of emotional episodes [12] to evaluate individual’s emotional competence, considering the psychological lexicon and the ability to attribute emotional mental states. Grazzani Gavazzi, Antoniotti, and Ornaghi [13] analyzed, in a sample of 14 years old pre-adolescents, the ability to mentalize anger and happiness. This sample has been compared with old adolescents (19 years old). The results indicated that older participants were able to consider anger and happiness in terms of mental states more than pre-adolescents, which in turn tended to use more terms related to behaviors. Other studies, especially in the field of clinical psychology, underlined the importance of parental mentalization for children’s socio-emotional development [3]. Parental responsiveness and mentalization exert an important joint effect on social learning and development in children. Thus, any disorder in these processes might hamper the development of these abilities [2]. 

Limits in parental mentalization might be a risk factor to develop a variety of psychological disorders, such as antisocial [14], borderline [15], and interpersonal problems like bullying and conduct problems during adolescence [16].

### 1.1. Bullying and Theory of Mind

Bullying is a phenomenon characterized by negative actions toward a peer, with the intention to hurt [17]. Bullying has been defined as a deliberate and ongoing misuse of power in relationships through verbal, physical, and/or social behavior that intends to cause physical, social, and/or psychological harm [18]. Bullying can involve an individual or a group misusing their power over one or more victims who feel unable to stop it from happening. Bullying can be obvious, with direct behaviors meant to hurt the victims (i.e., biting, kicking, stealing, or breaking personal objects, verbal threatening, treating one as a fool, being verbally mean) or hidden (i.e., social exclusion, isolation, manipulation).

Recent studies highlight the main cognitive and emotional mechanisms that characterize bullies. The model of the “Social skills deficit” [19] indicates that bullies show an atypical social information processing. Bullies tend to selectively code only certain kinds of social cues. They tend to apply some attributional bias when they interpret social information. Moreover, they show a reduced variety of responses to social conflicts [20].

Victims also show an atypical social information processing. Both bullies and victims describe “bullying” as some scenes that controls define simply as a playful fight. According to Crick e Dodge [21], it might be that bullies are less able to code harmful social information compared to non-bullies due to a lack of memory or selective attention. For this reason, they tend to attribute more negative intentions, produce less prosocial behaviors, predict more positive results from aggression. This kind of atypical information processing leads to more aggressive behaviors compared to non-bullies [22].

According to Lemerise and Arsenio [23], bullies’ interpretation of social cues is influenced by anger. The selection of the goals of actions is also influenced by anger or by a lack of empathy toward the victim. Anger characterizes reactive aggressive children [24]. While victims are only reactively aggressive [25], bullies are both proactively and reactively aggressive [26,27,28]. In victims, anger rises from the opinion that others are responsible for the negative behaviors toward them [28,29] due to previous experiences [30]. Other studies showed that bullies cannot empathize with victims. Therefore, they are unable to understand the emotional consequence of their behavior in others [31,32,33].

Bullies appear to not feel guilty for their actions possibly due to the mechanism of social disengagement [34]. The lack of empathy and the mechanisms of moral disengagement might hamper bullies to understand the cognitive perspective of the victim, but not their feelings. Indeed, bullies feel satisfied and proud of the personal advantages that they obtain by abusing the victim [35].

Sutton, Smith and Swettenham [36] indicated that not all bullies show difficulties in understanding others’ perspectives due to a lack of social information processing. Indeed, bullies use their ability to mentalize to obtain personal advantages. According to this approach, bullies are experts in social manipulation, so they could be defined as Machiavellian individuals [37], meaning that they can use their ability to read the minds to successfully harm the victims.

### 1.2. Mentalization of Emotions in Bullying

Several studies indicated significant differences in the ability to mentalize emotions in bullies, victims, and controls. Aggressive children are less accurate in identifying emotions compared to prosocial and altruistic classmates [38]. Particularly, bullies tend to attribute more likely the emotion of happiness to the assailant and they are not aware of the emotions of the victim. Moreover, bullies think that the victims deserve the attack. This means that bullies tend to focus more on their advantages than on the emotional effects on the victims. Some studies indicated that the inability to mentalize might influence the ability to inhibit violent actions [39]. Indeed, children with conduct disorders show low levels of mentalization [40]. Adults with antisocial disorder show difficulties in recognizing emotions and in social information processing, showing a hypo-mentalization [41]. Hypo-mentalization reflects an inability to consider complex models of mental states, with a consequent limited ability to understand others and self [42]. Hypo-mentalization might rise from reduced affective sensitivity of parents, due to various reasons like high levels of stress, anxiety or parental conflict [43,44]. Mentalization is also ineffective if it is dominated by automatic ideas excessively focused on the self, if it is guided by emotions or if it is excessively focused on others with ruminations [45].

Even though it is still not clear why a low level of mentalization is associated with a conduct disorder in children, it might be that an emotional dysregulation, particularly a predisposition to feel anger is associated with low levels of mentalization. Anger is associated with low self-awareness.

Studies of bully families indicate that domestic violence and family conflict, characterized by high emotional manipulation, coercion, and unpredictability, put children at risk of developing emotional dysregulation and impulsivity [46,47,48]. Difficulties in the capacity for emotional regulation could lead some children to maladaptive behavior in relationships with peers. Therefore, bullies learn to use anger in an instrumental and maladaptive way to reach their goals. In these situations, happiness might be associated mainly with the satisfaction to win a fight, which may lead to an increased perception of personal power [34,49]. However, the role of mentalization in these processes needs to be clarified [42]. Other studies indicated that victims’ families may have the opposite problem to that of bullies [50]: namely, a lack of familiarity with anger management and conflict situations.

Indeed, victims’ families are characterized by high levels of cohesion and overprotection [47,48], high levels of communication, and low levels of conflict and control. This could contribute to developing high levels of family dependency, fear of the outside world, social isolation, and difficulty relating to peers [51].

Since mentalization is a key ability in successful social interactions, bullies and victims might lack the ability to mentalize emotions like anger and happiness, maybe due to a difficult familiar culture, which tends to emphasize the expression of certain emotions and discourage the expression of others, with a detrimental effect on the awareness, the definition, and the narration of the emotions. Despite the relevance of the ability to mentalize anger and happiness for successful social relationships, no study so far, to the best of our knowledge, investigated this ability in bullies and victims.

### 1.3. Objective

Our study aims to fill this gap in the literature, by investigating possible differences between bullies, victims, and controls (students not involved in bullying in any role) in the ability to attribute mental states referred to the emotion of anger and happiness. Our hypothesis is that as compared to controls, bullies and victims will be less able to mentalize these emotions

## 2. Materials and Methods

### 2.1. Participants

This study involved 104 pre-adolescents in middle school, 44.2% males (*n* = 46) and 55.8% females *(n =* 58), age ranged between 13 years (89,4%; *n* = 93) and 14 years (10.6%; *n* = 11). Data collection took place in a suburban area of Cagliari (Italy), where the risks of school drop-out, psychological, and social malaise, drug abuse, and unemployment were high [52,53].

Data collection was done before the pandemic period of COVID-19. Parents gave written informed consent for the study. The study was approved by the local ethical committee of the Department of Pedagogy, Psychology, Philosophy of the University of Cagliari.

### 2.2. Procedure

To identify the bullies, the victims, and the comparison group, we applied The Olweus Bully/Victim Questionnaire [54].

We administered only the first two questions of the OBVQ aimed at classifying subjects as bullies, victims, and controls (Section 2.3).

To analyze the narrative episodes linked with the emotions of anger and happiness we applied a narrative approach [13], aimed to collect the description of the episodes associated with the emotions of our participants. The narrative approach used by Grazzani, Antoniotti, Ornaghi [13] focuses on techniques of the written narration of personal experiences [12] to elicit a personal reflection on his/her own emotional experiences. In this regard, we used the narrative tool of Grazzani, Antoniotti, and Ornaghi [13] to collect the narrator’s emotional episodes and his or her definitions of anger and happiness (see the section on measures).

The methodology used is based on the quantification of lexical forms concerning mental states, to document the increase in the ability to understand emotions. Previous research on this topic, conducted in the context of adolescence [13,55,56,57], showed the ability of adolescents to describe their emotions through mental verbs instead of behavioral verbs. The method of Montirosso, Pupino, and Borgatti [57] was used to distinguish mental predicates (verbs such as think, believe, feel) from behavioral predicates (verbs such as hit, run, smash). 

Coding was done by two independent researchers, with an inter-rater agreement of 92% (Cohen Kappa coefficient). Differences between categories were analyzed by a χ2 test with a level of significance of 0.05.

### 2.3. Measures

The Olweus Bully/Victim Questionnaire (OBVQ) [54] is an anonymous self-report questionnaire and it is one of the most widely used instruments to measure the prevalence of bullying worldwide [17]. The original version of the OBVQ was developed in 1983 (with 36 items), and in 1996, Dan Olweus put forward the revised questionnaire (OBVQ-R) and increased the number of items to 42. The revised version has a more specific criterion of frequency: the response option “sometimes” in the original version was changed to “2 or 3 times a month” [58].

Several studies showed evidence of the validity of the OBVQ using different methodological approaches [58]. The results supported the validity and reliability of the OBVQ-R to identify two main categories: being bullied and bullying others.

The questionnaire starts with two global questions where students could identify themselves as victims or bullies: “How often have you been bullied at school in the past couple of months?” (victims), and “How often have you bullied at school in the past couple of months?” (perpetrators). 

The answers can be coded on a five-point scale from 0 to 4 (0 = it has not happened to me in the last two months, 1 = it happened to me only once or twice, 2 = it happened to me 2 to 3 times a month, 3 = it happened to me once a week, 4 = it happened to me several times a week) [58].

Children who had been bullied “2 to 3 times a month or more” were classed as victims. Those who had bullied others “2 to 3 times a month or more” were classed as bullies. Then, children who had both bullied others, and been bullied, at least “2 to 3 times a month or more” as “bully-victim”. Whereas those who had responded that they “have not” been bullied/not bullied other students or have been bullied/have bullied other students “only once or twice’’ are considered “non-victims or non-bullies” [58]. Those last were often considered as a comparison group (controls) because they are not involved in bullying in any role [54,58].

The narrative tool of Grazzani, Antoniotti, and Ornaghi [13] includes four items.

This approach includes four open-ended questions, as follows:

1. Describe an episode of your life in which you have been angry.
2. Describe what is anger for you.
3. Describe an episode of your life in which you have been happy.
4. Describe what is happiness for you.

We coded the participants’ answers according to the method used by Montirosso, Pupino, and Borgatti [57], which allows us to identify the specific terms used by the participants to describe the emotions. In line with this method, we counted the “total number of words used to define happiness”, the “total number of words used to define anger”, the “mental states predicates related with happiness”, “the behavioral predicates related to happiness”, “the mental states predicates related to anger” and “the behavioral predicates related with anger”. The behavioral predicates also included the physical states. 

## 3. Results

The results indicated that bullies were 25% *(n =* 26), victims were 15.4% *(n =* 16), bullies-victims 3.8% *(n =* 4) and students “non-victims or non-bullies” (controls) were 55.8% *(n =* 58). In our analysis, we did not compute the answers of the bullies–victims, which were very rare *(n =* 4), and which have been considered in previous studies as a source of high variability [59]. Analyses carried out by gender, with the Olweus Bully-Victim Questionnaire (in the two global questions where students could identify themselves as victims or bullies) showed that bullies were 53.8% males and 46.1% females (χ^2^ = 0.288; α > 0.05; ns). Victims were 43.75% males and 56.25% females (χ^2^ = 0.78; α > 0.05; ns) and controls were 48.3% males and 51.7 females (χ^2^ = 0.057; α > 0.05; ns). Age was not considered as a significant variable because all students who were interviewed were in third year of secondary school and were between 13 and 14 years old. Among the invalid questionnaires (poorly completed or incomplete), those of four students over the age of 14 were eliminated and were not considered part of the overall sample.

### 3.1. Results of Anger

#### 3.1.1. Episodes Related to Anger

Here, we indicated the results of the first item, which asked the participants to describe an episode related to anger, in the three groups of bullies, victims, and controls (Table 1).

The category indicated by bullies as most associated with anger was “problems with classmates” (37.1%). The category indicated by victims and controls as most associated with anger was instead “problems with friends” (28.7% vs. 32.5%; χ^2^ = 3.04, α > 0.05, ns). As compared to the control group far more bullies reported problems with classmates (37.1% vs. 18.9%; χ^2^ = 22.67, α ≤ 0.05). On the contrary, as compared to controls, fewer victims reported having problems with their classmates (7.1% vs. 18.9%; χ^2^ = 56.15, α ≤ 0.05). It seems that, within the context of the social relationships with classmates, bullies experience more anger compared with victims and controls. Victims tend to feel less angry compared with bullies and with peers not involved in bullying (controls). It might be of interest to explore in detail how the three groups mentalize anger.

#### 3.1.2. Anger as a Mental State in the Bullies

Here, we indicated the definitions of anger in the bullies and in the control group, in the second item that investigated the ability to mentalize anger (Table 2).

As compared to controls, fewer bullies attributed anger to mental states (19.2% vs. 38.6%; χ^2^ = 21.57; α ≤ 0.05). 

Bullies tended to define anger mainly in terms of behavior or physical state (34.6% yes 26.3%; χ^2^ = 15.97, α ≤ 0.05) and as “thing or moment” (30.8% vs. 24.6%; χ^2^ = 20.27, α ≤ 0.05), compared to controls. 

Below are some examples of the definition of anger in bullies:

- “Anger is when I feel like smashing everything” (anger as a thing or moment);
- “When someone makes me angry, I want to hit him” (anger as behavior state);
- “When I’m angry I feel like bursting” (anger as a behavioral state);
- “Anger is an emotion that occurs when I believe others want to boss me around” (anger as a mental state).

Below is an example of the definition of anger in a student not involved in bullying (controls):

- “Anger is an emotion that makes me believe I am right when I believe I am experiencing injustice”.

As you can see, there are many references to mental states. These results as a whole indicate a bullies’ difficulty in considering anger as a mental state.

#### 3.1.3. Anger as a Mental State in the Victims

Here, we indicated the definitions of anger in the victims and in the control group, in the second item that investigated the ability to mentalize anger (Table 3).

As compared to controls, fewer victims considered anger in terms of mental state (12.5% vs. 38.6%; χ^2^ = 30.72, α ≤ 0.05). Victims considered anger more in terms of behavior or physical state (37.5% vs. 26.3%; χ^2^ = 14.35, α ≤ 0,05) or as “thing or moment” (31.3% vs. 24.6%; χ^2^ = 19.89, α ≤ 0.05), compared to controls. Below are some examples of the definition of anger in victims:

- “Anger is an ugly thing” (anger as a thing or moment);
- “Anger leads me to distance myself from certain companions” (anger as behavior state);
- “I think it’s not right to feel angry” (anger as a mental state).

These results also indicate a victims’ difficulty in considering anger as a mental state.

#### 3.1.4. Lexicon Related to Anger

Here, we indicated the number of terms that bullies, controls and victims use to refer to anger, expressed in terms of percentage (Table 4), in response to the second item (“Describe what anger means for you”).

Bullies used a significantly lower number of terms referred to as anger (50%) in the category (4–14) compared to controls (χ^2^ = 6.48, α ≤ 0.05). Controls used mainly a moderate number of terms related to mental states (44%), in the range (15–25). The victims did not differ compared with controls in the number of terms used to refer to anger (χ^2^ = 0.72, α > 0.05, ns), since they used the 50% of terms in the intermediate category (15–25 words) compared with controls (44%).

#### 3.1.5. Summary about Anger

Bullies and victims show the same difficulty in mentalizing emotions, considering anger mainly as a behavior or physical state. However, victims are different than bullies, since they use more terms related to anger, which might indicate a higher ability to talk about anger compared with bullies. If this is true, we should find the same results for happiness. 

### 3.2. Results about Happiness

#### 3.2.1. Episodes Associated with Happiness

Here, we indicated the results of the third item, which asked the participants to describe an episode related to happiness, in the three groups of bullies, controls, and victims (Table 5).

Bullies associated less happiness with “presents, parties and travels” (18.5% vs. 37.8%; χ^2^ = 22.82, α ≤ 0.05) compared with controls. They also claimed to be “rarely happy” (vs 11.1% vs. 5.4%; χ^2^ = 70.05, α ≤ 0.05) in their interactions with parents and friends (25.9% vs. 32.5%; χ^2^ = 17.74, α ≤ 0.05) compared with controls.

Victims, like bullies, tended to associate less happiness to “presents, parties and travels” (21% vs. 37.8%; χ^2^ = 19.80, α ≤ 0.05) compared to controls.

#### 3.2.2. Happiness as a Mental State in the Bullies

Here, we indicated the definitions of happiness in the bullies and in the control group in the fourth item, which investigated the ability to mentalize happiness (Table 6).

As compared to controls, fewer bullies attributed happiness to mental states (15.4% vs. 37.9%; χ^2^ = 26.87, α ≤ 0.05). Bullies tended to refer more to happiness as a “behavior or physical state” (34.6% vs. 22.4%; χ^2^ = 19.97, α ≤ 0.05) and as a “thing or moment” (26.9% vs. 19%; χ^2^ = 29.89, α ≤ 0.05) compared to controls. 

Below are some examples of the definition of happiness in bullies:

- “Happiness is something that rarely happens to me” (happiness as a thing).
- “When I’m happy I feel like screaming” (happiness as behavior state).
- “I think I’m happy when others respect me” (happiness as a mental state).

These results indicate bullies’ difficulty in considering happiness as a mental state.

#### 3.2.3. Happiness as a Mental State in the Victims

Here, we indicated the definitions of happiness in the victims and in the control group, in the fourth item, which investigated the ability to mentalize happiness (Table 7).

As compared to controls, fewer victims considered happiness in terms of mental state (25% vs. 37.9%; χ^2^ = 15.42, α ≤ 0.05). Victims considered happiness more in terms of behavior or physical state (31.3% vs. 22.4%; χ^2^ = 22.22, α ≤ 0.05), compared to controls.

Below are some examples of the definition of happiness in victims:

- “Being happy is a great thing!” (happiness as thing or moment);
- “When I’m happy I want to tell everyone” (happiness as behavior state);
- When I’m happy I feel light” (happiness as a physical state);
- “When I’m happy, I think everything will be fine” (happiness as mental state).

#### 3.2.4. Lexicon Related to Happiness

Here, we indicated the number of terms that bullies, controls, and victims use to refer to happiness, expressed in terms of percentage (Table 8), in response to the fourth item (“Describe what is happiness for you”). 

Most bullies used very few terms to refer to happiness (2–12) compared to controls (41% vs. 20%; χ^2^ = 19.62, α ≤ 0.05). Most of the of the controls (40%) used an intermediate number of terms (13–23), while most victims (40%) used a significantly higher number of terms (χ^2^ = 10.30, α ≤ 0.05) to refer to happiness, compared to controls (30%). 

#### 3.2.5. Summary of the Results about Happiness

Both bullies and victims show difficulties in defining happiness in terms of mental states, focusing mainly on behavior and physical states compared to controls. However, victims tend to use a higher number of terms to define happiness, which might indicate that they are more able to talk about emotions. Bullies use a very low number of terms to define happiness. It is possible that, even though bullies and victims share some level of difficulties in referring to mental states, bullies might be more resistant to talking about emotions.

## 4. Discussion

This study investigated how pre-adolescents mentalize the emotions of anger and happiness. The results indicated atypical social cognition both in bullies and victims but not in controls. Specifically, bullies associate anger mainly with socio-relational problems with their classmates. On the contrary, victims rarely associated anger with these problems, with a lower frequency compared with controls. It seems that bullies get angry too much in the interactions with their classmates, while victims get angry very rarely. These results are in line with previous studies, indicating an atypical perception of anger both in bullies and victims [23,30].

Morosan, Fonseca-Pedrero, Debbane [16] interpreted this phenomenon by indicating that bullies are not able to mentalize anger and therefore tend to use it in non-functional and instrumental ways with their peers. This is in line with the lower number of terms that bullies use to refer to anger compared to controls. Additionally, it is in line with the higher number of behavioral and physical terms that they use to refer to this emotion. Taken together, these results might indicate a “hypo-mentalization” in bullies.

Previous studies indicated that the phenomenon of hypo-mentalization might arise from an inability of the parents to be emotionally attuned to their children, maybe due to higher levels of stress and/or familial conflict [16,42]. Previous experiences within their familiar and cultural context might lead the bullies to express anger and happiness, especially in response to winning a fight, rather than for the mere pleasure associated with an intimate social relationship [49]. 

Our results shows that victims have the same difficulty in metalizing anger. They tend to refer to this emotion in terms of a “thing” or a “moment”. It is possible that victims learned to avoid anger with the classmates, probably because it is perceived as dangerous outside of their family. Previous studies on the familiar background of the victims [47,48,51] showed higher levels of hyper protection compared to the familiar background of bullies. Victims also show a higher tendency of internalizing problems rather than externalizing ones, which in turn is more common in bullies [59,60]. The higher tendency of the victims to talk about happiness might instead be further investigated. It could be due to a tendency to avoid negative emotions (hyper protection) compared to the families of the controls. However, this tendency could be misunderstood by bullies, who in their families tend to express happiness in a colder way [46,47,48]. The role of mentalization in these processes therefore needs to be further investigated.

From this point of view, our study has some limitations. First, it is necessary to obtain additional data about the socioeconomic and family background of bullies and victims to better understand some of the findings. It would also be interesting in future research to use other quantitative tools to explore the role of other important emotions such as fear and shame in victims and guilt in bullies. 

In summary, the results of our study integrate the data already present in the literature, which highlights difficulties in mentalizing anger in bullies and victims [16,24,25,28]. Our results also confirm what was found in previous studies about the development of emotions in adolescence [13,56,57]. In fact, in line with these studies [13,56,57], we found that adolescents not involved in bullying are characterized by a “mental” knowledge of their emotions. On the contrary, preadolescents bullies and victims showed a “behavioral” understanding of their emotions, likely attributable to immaturity in emotional development [48,49]. Our study is different from previous ones for several reasons. First, we focused on two specific emotions, anger and happiness, and not on all emotions in general. Second, while previous studies on the Theory of Mind in bullies and victims [19,20] focused mainly on epistemic mental states, we investigated non-epistemic mental states like emotions. Third, even though few previous studies highlighted that anger and empathy play a fundamental role in the processing of social information in bullies and victims [23,31], our study extends these results by using a narrative approach. This approach is easy to use in the educational settings and it might have important implications for possible intervention at school to promote social–emotional competencies in pre-adolescents, especially in those at risk of bullying.

## 5. Conclusions

This contribution has practical implications for prevention of distress in preadolescents and promotion of well-being at school. It is necessary, therefore, to apply practical guidelines in terms of addressing the bullies’ and victims’ limitations of mentalizing emotions, trough the socio-affective education at school [61]. For victims, we must consider programs specifically aimed at making them aware of emotions, beliefs and relational tactics. Mentalizing emotions is important for victims to recognize the emotional signals of anger in relationships with bullies and peers and to develop assertiveness [62].

For bullies, some preventive measures may be created to promote awareness of anger and teach them how to handle aggressive behavior and empathize with their peers.

The results of our study indicate the need to define effective intervention programs to prevent bullying by promoting adequate mentalizing emotions. 

The construct of the mentalization is essential to understand the development of the ability to regulate emotions in preadolescence, promoting the reflective function of the mind [63]. Finally, we highlight the need to train teachers [63] and parents [2] about the importance of awareness of emotions to be understood as a valuable “ally” of the cognitive and social processes involved in school and family education.

## Figures and Tables

**Table 1:** Percentages of the different categories of episodes related to anger in response to the first item in the three groups of bullies, controls, and victims.

Episodes Related to Anger	Bullies %	Controls %	Victims %
Conflict with parents	7.4	8.1	21.4
School failure	7.4	13.5	7.1
Problems with friends	7.4	32.5	28.7
Problems with classmates	37.1	18.9	7.1
Other	14.8	21.6	21.4
No answers	25.9	5.4	14.3
Total	100	100	100

Item 1: “Describe an episode of your life in which you have been angry”.

**Table 2:** Percentage of lexicon referred to the definition of anger in bullies and controls.

Definition of Anger	Bullies %	Controls %
Feeling or emotion	19.2	38.6
Behavior or physical state	34.6	26.3
Thing or moment	30.8	24.6
Other	15.4	10.5
Total	100	100

Item 2: “Describe what is anger for you”.

**Table 3:** Percentage of lexicon referred to the definition of anger in victims and controls.

Definition of Anger	Victims %	Controls %
Feeling or emotion	12.5	38.6
Behavior or physical state	37.5	26.3
Thing or moment	31.3	24.6
Other	18.3	10.5
Total	100	100

Item 2: “Describe what is anger for you”.

**Table 4:** Percentages of the number of terms related to anger in response to the second item in the three groups of bullies, controls, and victims.

Number of Terms Used to Refer to Anger	Bullies %	Controls %	Victims %
4–14	50	32	21
15–25	23	44	50
26–36	27	24	29
Totale	100	100	100

Item 2: “Describe what is anger for you”.

**Table 5:** Percentages of the different categories of episodes related to happiness in response to the third item in the three groups of bullies, controls, and victims.

Episodes Related with Happiness	Bullies %	Controls %	Victims %
For a present, a party or a travel	18.5	37.8	21
When I feel fine with my parents and friends	25.9	32.5	32.5
When I play sport	11.1	5.4	7
I’m rarely happy	11.1	5.4	7
Other	14.8	13.5	25.5
No answer	18.6	5.4	7

Item 3: “Describe an episode of your life in which you have been happy”.

**Table 6:** Percentage of lexicon referred to the definition of happiness in bullies and controls.

Definition of Happiness	Bullies %	Controls %
Feeling or emotion	15.4	37.9
Behavior or physical state	34.6	22.4
Thing or moment	26.9	19.0
Other	23.1	20.7
Total	100	100

Item 4: “Describe what is happiness for you”.

**Table 7:** Percentage of lexicon referred to the definition of happiness in bullies and controls.

Definition of Happiness	Victims %	Controls %
Feeling or emotion	25.0	37.9
Behavior or physical state	31.3	22.4
Thing or moment	18.8	19.0
Other	25.0	20.7
Total	100	100

Item 4: “Describe what is happiness for you”.

**Table 8:** Percentages of terms related to happiness in response to the fourth item in the three groups of bullies, controls, and victims.

Number of Terms Used to Refer to Happiness	Bullies %	Controls %	Victims %
2–12	41	20	26
13–23	27	40	44
24–34	32	40	30
Total	100	100	100

Item 4: “Describe what is happiness for you”.

## Data Availability

Upon reasonable request, data used and analyzed during the current study are available from the corresponding author.

## References

[1] Bateman A.W., Fonagy P. (2012). Handbook of Mentalizing in Mental Health Practice.

[2] Sharp C., Shohet C., Givon D., Penner F., Marais L., Fonagy P. (2020). Learning to mentalize: A mediational approach for caregivers and therapists. Clin. Psychol. Sci. Pract..

[3] Fonagy P., Gergely G., Jurist E., Target M. (2002). Affect Regulation, Mentalization and the Development of the Self.

[4] Battistelli P. (1992). La Rappresentazione Della Soggettività.

[5] Wimmer H.M., Perner J. (1983). Beliefs about Beliefs: Representation and Constraining Function of Wrong Beliefs in Young Children’s Understanding of Deception. Cognition.

[6] Bloom P., German T. (2000). Two reasons to abandon the false believe task as a test of theory of mind. Cognition.

[7] Hughes C., Leekam S. (2004). What are the links between theory of mind and social relations? Review, reflections and new directions for studies of typical and atypical development. Soc. Dev..

[8] Saarni C. (1999). The Development of Emotional Competence.

[9] Pons F., Harris P.L., De Rosnay M. (2004). Emotion comprehension between 3 and 11 years: Developmental period and hierarchical organization. Eur. J. Dev. Psychol..

[10] Pons F., Harris P., Doudin P.A. (2002). Teaching emotion understanding. Eur. J. Psychol. Educ..

[11] Grazzani Gavazzi I., Ornaghi V., Pirali F. (2011). Teoria della mente e comprensione del lessico psicologico nei bambini: Dati preliminari di validazione del Test di Lessico Emotivo. Psicol. Clin. Dello Svilupp..

[12] Atkinson R. (1998). The Life Story Interview. Qualitative Research Methods.

[13] Grazzani Gavazzi I., Antoniotti C., Ornaghi V. (2008). Emozioni e adolescenza: Proposte di uno strumento di indagine, primi risultati e possibili applicazioni in ambito educativo. Psicol. Clin. dello Svilupp..

[14] Bateman A., Bolton R., Fonagy P. (2013). Antisocial personality disorder: A mentalizing framework. Am. Psychiatr. Publ..

[15] Fonagy P., Luyten P. (2009). A developmental, mentalization-based approach to the understanding and treatment of borderline personality disorder. Dev. Psychopathol..

[16] Morosan L., Fonseca-Pedrero E., Debbane M. (2020). Network Analysis of Reflective Functioning and Conduct Problems During Adolescence. Psychol. Violence.

[17] Olweus D. (1993). Bullying at School. What We Know and What We Can Do.

[18] Smith P.K., Kwak K., Toda Y. (2016). School Bullying in Different Cultures.

[19] Dodge K.A. (1980). Social cognition and children’s aggressive behavior. Child. Dev..

[20] Dodge K.A., Perlmutter M. (1986). A social information processing model of social competence in children. The Minnesota Symposium on Child Psychology.

[21] Crick N.R., Dodge K.A. (1994). A review and reformulation of social information-processing mechanisms in children’s social adjustment. Psychol. Bull..

[22] Dodge K.A., Crick N.R. (1990). Social information- processing bases of aggressive behavior in children. Pers. Soc. Psychol. Bull..

[23] Lemerise E.A., Arsenio W.F. (2000). An integrated model of emotion processes and cognition in social information processing. Child. Dev..

[24] Crick N.R., Dodge K.A. (1996). Social information processing mechanisms in reactive and proactive aggression. Child. Dev..

[25] Dodge K.A., Lochman J.E., Harnish J.D., Bates J.E., Pettit G.S. (1997). Reactive and proactive aggression in school children and psychiatrically impaired chronically assaultive youth. J. Abnorm. Psychol..

[26] Camodeca M., Goossens F.A., Meerum Terwogt M., Schuengel C. (2002). Bullying and victimization among school-age children: Stability and links to proactive and reactive aggression. Soc. Dev..

[27] Camodeca M., Goossens F.A., Schuengel C., Meerum Terwogt M. (2003). Links between social information processing in middle childhood and involvement in bullying. Aggress. Behav..

[28] Camodeca M., Goossens F.A. (2005). Aggression, social cognitions, anger and sadness in bullies and victims. J. Child. Psychol. Psychiatry.

[29] Graham S., Hudley C., Williams E. (1992). Attributional and emotional determinants of aggression among African-American and Latino young adolescents. Dev. Psychol..

[30] Arsenio W.F., Lemerise E.A. (2001). Varieties of childhood bullying: Values, emotion processes, and social competence. Soc. Dev..

[31] Gini G. (2006). Social cognition and moral cognition in bullying: What’s wrong?. Aggress. Behav..

[32] Gini G., Albiero P., Benelli B., Altoè G. (2007). Does empathy predict adolescents’ bullying and defending behavior?. Aggress. Behav..

[33] Endresen I.M., Olweus D., Bohart A.C., Stipek D.J. (2001). Self-reported empathy in Norwegian adolescents: Sex differences, age trends, and relationship to bullying. Constructive & Destructive Behavior: Implications for Family, School, & Society.

[34] Bandura A. (2015). Moral Disengagement: How People Do Harm and Live with Themselves.

[35] Menesini E., Fonzi A., Sanchez V. (2002). Attribuzioni di emozioni di responsabilità e disimpegno morale in una storia di bullismo. Differenze tra bulli, vittime, esterni e difensori. Età Evol..

[36] Sutton J., Smith P.K., Swettenham J. (1999). Bullying and “theory of mind”: A critique of the “social skills deficit” view of anti-social behaviour. Soc. Dev..

[37] Sutton J., Keogh E. (2000). Social competition in school: Relationships with bullying, Machiavellianism and personality. Br. J. Educ. Psychol..

[38] Fonzi A. (1999). Il Gioco Crudele: Studi e Ricerche Sui Correlati Psicologici del Bullismo.

[39] Möller C., Falkenström F., Mattias H.L., Holmqvist R. (2014). Mentalizing in young offenders. Psychoanal. Psychol..

[40] Donahue J.J., Goranson A.C., McClure K.S., Van Male L.M. (2014). Emotion dysregulation, negative affect, and aggression: A moderated, multiple mediator analysis. Pers. Individ. Differ..

[41] Newbury-Helps J., Feigenbaum J., Fonagy P. (2017). Offenders with antisocial personality disorder display more impairments in mentalizing. J. Pers. Disord..

[42] Ferreras Picazo M., Cormenzana Redondo S., Martínez-Pampliega A. (2021). Interparental Conflict and Bullying: Assessment of the Mediating Role of Mentalization and Emotion Regulation. Rev. Iberoam. Diagn. Eval.-E.

[43] Davies P.T., Cummings E.M. (1994). Marital conflict and child adjustment: An emotional security hypothesis. Psychol. Bull..

[44] Lyons-Ruth K., Bronfman L., Atwood G., Solomon J., George C.C. (1999). A relational diathesis model of hostile helpless states of mind: Expressions in mother-infant interaction. Attachment Disorganization.

[45] Fonagy P., Luyten P., Moulton-Perkins A., Lee Y., Warren F., Howard S., Lowyck B. (2016). Development and validation of a self- report measure of mentalizing: The reflective functioning questionnaire. PLoS ONE.

[46] Davies P.T., Martin M.J. (2013). The reformulation of emotional security theory: The role of children’s social defense in developmental psychopathology. Dev. Psychopathol..

[47] Bowers L., Smith P.K., Binney V. (1994). Perceived Family Relationships of Bullies, Victims and Bully/Victims in Middle Childhood. J. Soc. Pers. Relatsh..

[48] Cook C.R., Williams K.R., Guerra N.G., Kim T.E., Sadek S. (2010). Predictors of bullying and victimization in childhood and adolescence: A meta-analytic investigation. Sch. Psychol. Q..

[49] Fonzi A., Ciucci E., Berti C., Brighi A. (1996). Riconoscimento delle emozioni, stili educativi familiari e posizioni nel gruppo in bambini che fanno e subiscono prepotenze a scuola. Età Evol..

[50] Finnegan R.A., Hodges E.V.E., Perry D.G. (1998). Victimization by peers: Associations with children’s reports of mother–child interaction. J. Pers. Soc. Psychol..

[51] Hawker D.S.J., Boulton M.J. (2000). Twenty Years’ Research on Peer Victimization and Psychosocial Maladjustment: A Meta-Analytic Review of Cross-Sectional Studies. J. Child. Psychol. Psychiatry Allied Discip..

[52] Ministero Dell’istruzione-Ufficio Gestione Statistica La Dispersione Scolastica aa.ss. 2017/2018–2018/2019–2019/2020. https://www.miur.gov.it/documents/20182/0/La+dispersione+scolastica+aa.ss.2018-2019+e+aa.ss.2019-2020.pdf/99ea3b7c-5bef-dbd1-c20f-05fed434406f?version=1.0&t=1622822637421.

[53] Pedditzi M.L., Lucarelli L. Direct bullying at school and depressive risk in Early Adolescence. Proceedings of the European Proceedings of Social & Behavioural Science 2019, 9th International Conference on Education and Educational Psychology.

[54] Olweus D. (1996). The Revised Olweus Bully/Victim Questionnaire.

[55] Grazzani Gavazzi I. (2004). La competenza emotiva. Studi e ricerche nel ciclo di vita.

[56] Confalonieri E., Scaratti G., Tomisich M. (1998). L’autobiografia come possibile strumento di valutazione. La costruzione del sé negli adolescenti. Uno studio pilota, Archivio di Psicologia. Neurol. E Psichiatr..

[57] Montirosso R., Pupino C., Borgatti R., Grazzani Gavazzi I. (2004). Mentalizzazione delle emozioni in adolescenza: Analisi dei processi narrativi. La Competenza Emotiva. Studi e Ricerche Nel Ciclo di Vita.

[58] Solberg M.E., Olweus D. (2003). Prevalence estimation of school bullying with the Olweus Bully/Victim Questionnaire. Aggress. Behav..

[59] Copeland W.E., Wolke D., Angold A., Costello E.J. (2013). Adult Psychiatric Outcomes of Bullying and Being Bullied by Peers in Childhood and Adolescence. Jama Psychiatry.

[60] Pedditzi M.L., Lucarelli L. (2014). Bullying and depressive risk: An exploratory survey in a sample of students in early adolescence. Med. Bambino.

[61] Murray D.W., Rosanbalm K. (2017). Promoting Self-Regulation in Adolescents and Young Adults: A Practice Brief.

[62] Pedditzi M.L., Nonnis M., Massidda D. (2016). Assertività e soddisfazione degli studenti nelle transizioni scolastiche: Una ricerca nella scuola secondaria superiore. Psicologia della Salute.

[63] Damiani P. (2011). Theory of mind and reflective function Identification of an interdisciplinary field of study for teacher trainings. Form. E Insegn..

